# Appendiceal Diverticulitis Presenting as Acute Appendicitis: A Case Report

**DOI:** 10.7759/cureus.32626

**Published:** 2022-12-17

**Authors:** Mostafa Elkhawaga, Basavaraj Mundasad, Jacob Hampton, Ahmad S Alam

**Affiliations:** 1 General Surgery, John Hunter Hospital, Newcastle, AUS; 2 General Surgery, Armidale Hospital, Armidale, AUS

**Keywords:** appendix, appendiceal diverticulitis, histopathology (hp), right iliac fossa (rif) pain, appendiceal diverticulosis

## Abstract

Appendiceal diverticulitis (AD) is an overlooked pathology that carries a high risk of perforation and associated neoplasia, especially carcinoid tumours and mucinous adenoma. AD may be congenital, but more commonly acquired. It may cause diverticulitis, which causes clinical and radiological signs like those of acute appendicitis, and that may delay the diagnosis till it is confirmed on histopathological examination. Here we report a case of acute AD in a case initially diagnosed as acute appendicitis.

## Introduction

Appendiceal diverticulitis (AD) is a rare pathology that was reported for the first time by Kelynack in 1893 [1]. AD has a variable incidence in the literature ranging from 0.004% to 3.7% [1-4]. The documented incidence of AD may be underestimated due to the clinical and radiological overlap between it and acute appendicitis [5]. AD is considered one of the differential diagnoses in cases presented with lower abdominal pain or in cases with suspected appendicitis. Roughly two out of three patients with AD develops diverticulitis [6].

## Case presentation

A 71-year-old male presented to the emergency department with right iliac fossa pain for less than 24 hours with no associated fever, vomiting, or diarrhoea. His past medical/surgical history includes bilateral inguinal hernias repair, umbilical hernia repair, radical prostatectomy for prostatic cancer, and tonsillectomy. On examination, the patient was hemodynamically stable, with a blood pressure of 131/78 mmHg and a heart rate of 76 beats per minute. His temperature was noted to be 36.5°C and he was saturating at 97% on room air. His abdominal examination showed right iliac fossa tenderness and rebound tenderness, but no clinical signs of peritonitis.

He underwent a series of laboratory tests that have been detailed in Table 1. The results showed mildly elevated white cell count (WCC), neutrophils, and C-reactive protein (CRP). His liver function tests and kidney function tests were unremarkable and within normal limits.

**Table 1:** Laboratory investigations at the time of presentation.

Laboratory investigations	Results	Normal range
White cell count	12 x10^9^/L	4-11 x10^9^/L
Neutrophils	10.8 x 10^9^/L	2-8 x10^9^/L
Haemoglobin	156 g/L	130-180 g/L
C-reactive protein	7 mg/L	<5 mg/L
Bilirubin	18 umol/L	<20 umol/L
Creatinine	100 umol/L	60-110 umol/L

**Figure 1:**
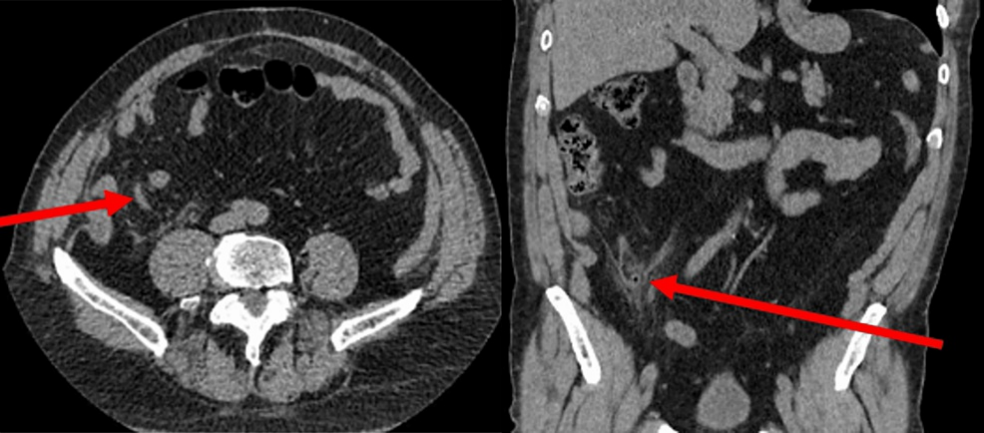
Preoperative computed tomography showing acute uncomplicated appendicitis.

**Figure 2:**
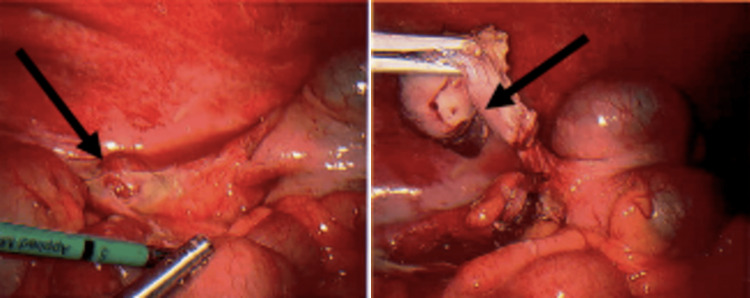
Intraoperative pictures of acute appendicitis.

The patient underwent a computed tomography (CT) scan, which revealed large bowel diverticulosis and acute tip appendicitis with no evidence of perforation or abscess formation (Figure 1), which was in keeping with the clinical diagnosis. The patient was offered a diagnostic laparoscopy and appendicectomy. Informed consent was obtained, and the patient went through a diagnostic laparoscopy and appendicectomy without immediate intraoperative complications. The intraoperative findings include a grossly inflamed appendix, adherent to the abdominal wall (Figure 2).

Laparoscopic appendicectomy and a peritoneal examination were carried out. The specimen was sent for histopathological examination. The postoperative course was uneventful, and the patient was discharged home the next day in optimal condition with a follow-up plan in two weeks to check on the histopathology results. In the follow-up appointment, the patient was asymptomatic with no complications.

**Figure 3:**
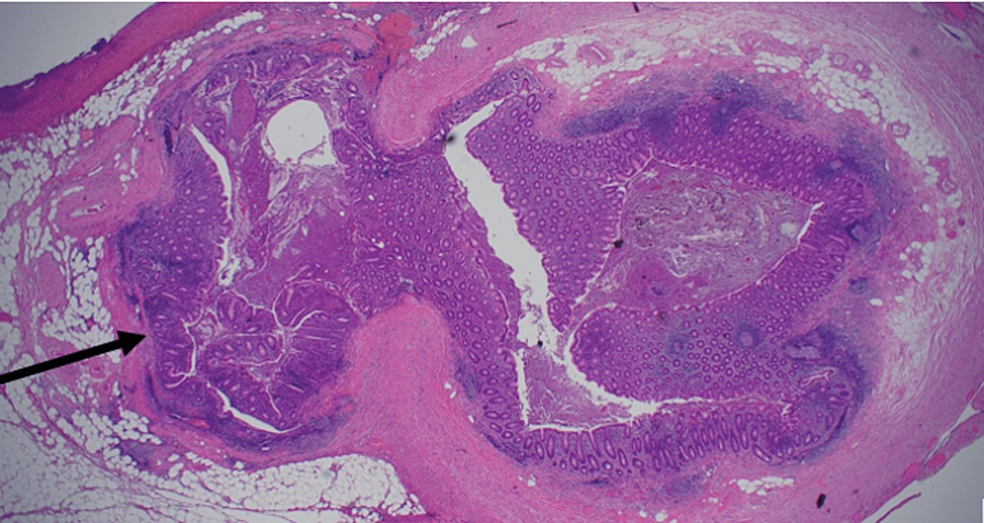
Histopathological image showing appendiceal diverticulitis.

The histopathological examination showed macroscopic findings of a heavily inflamed appendix measuring 62 mm in length and up to 9 mm in diameter with an attached fibrosed mesoappendix measuring 39x16x19 mm^3^. On the serosal surface and mesoappendix, there were some fibrinous adhesions and fibrinopurulent exudate. Cut sections revealed the distal tip filled with faecal material with fibrinopurulent exudate. The microscopic histopathological findings included features of acute appendicitis and foci of ulceration and acute transmural inflammation arising from the wall at the site of the AD. There was no evidence of dysplasia or malignancy (Figure 3).

## Discussion

AD is one of the rare causes of right iliac fossa pain/acute abdomen, which mimics acute appendicitis. The mean age of patients with AD is higher than that of acute appendicitis with more predominance in males than females [1]. A retrospective study done on 1586 patients who had appendectomies showed that the mean age for AD was 34.4 years with a male-to-female ratio of 4:1 [4]. It may be congenital or acquired, true or false, and can be divided (on a pathological basis) into four types depending on the inflammation of the diverticulum and appendix as detailed in Table 2 [7]. Our case was type 2 given the histopathology report showed acute appendicitis and transmural inflammation arising from the wall at the site of AD.

**Table 2:** Pathological types of appendiceal diverticulitis.

	Appendiceal diverticulitis	Appendix
Type 1	Inflamed	Non-inflamed
Type 2	Inflamed	Inflamed
Type 3	Non-inflamed	Inflamed
Type 4	Non-inflamed	Non-inflamed

Classification into congenital and acquired types is based on the defective layers of the appendiceal wall [1]. Acquired AD is more common than congenital AD. The congenital type is a true diverticulum that occurs because of an abnormal congenital defect, compromising all wall layers (mucosa, submucosa, muscular and serosa) and found to occur on the anti-mesenteric border [8,9]. The acquired type is a false diverticulum which occurs as a result of a defect in the muscular layer of the appendiceal wall through which the mucosa and submucosa usually bulge and form a false AD. The false acquired AD usually occurs on the mesenteric border and has a higher perforation risk than congenital AD [10,11]. The pathogenesis of acquired AD is not well understood but the risk factors include colonic diverticulosis and other appendiceal pathologies [12-15], age over 30, male gender, Hirschsprung’s disease, and cystic fibrosis (CF) [2]. In our case, the CT shows uncomplicated colonic diverticular disease. The pathogenesis of congenital AD is not well understood, but it may be related to genetic defects such as D13-15 trisomy syndrome [16].

AD can present as an incidental finding, diverticulitis, or acute appendicitis. There is a clinical overlap between AD and acute appendicitis, but right iliac fossa pain in older age for a longer period of time is suspicious for AD [1,3]. Perforation is more common in AD than in appendicitis. The perforation rate of AD is varying in different literature; 66% (four times greater than the perforation rate in acute appendicitis) [7], 33% [13], and 65.8% (six times greater than the perforation rate in acute appendicitis) [3]. AD is commonly associated with neoplasms, especially carcinoid and mucinous neoplasms [17]. Two percent of patients (in a study including 57 patients with ADs) were found to have neoplasia. The rate of appendiceal neoplasia is 10 times more in people with AD than those without AD [18]. There was no evidence of perforation or associated neoplasm in the case discussed.

Identification of AD on CT can be difficult because of the associated inflammatory changes [19]. Histopathological diagnosis is very important to follow as the clinical and radiological differentiation between AD and acute appendicitis is difficult [1,4,20]. Our case was only a histopathological diagnosis.

## Conclusions

In conclusion, AD is a rare and dangerous pathology that needs to be considered on assessing a patient with suspected appendicitis. It is impossible to differentiate between AD and appendiceal neoplasia based on clinical or radiological assessment. Because of this, we recommend appendicectomy and accurate histopathological examination, whenever it is difficult to differentiate between AD and other pathology, to avoid missing a sinister pathology.
